# The Significance of Elevated sST2 in Children with Kawasaki Disease

**DOI:** 10.3390/children12070868

**Published:** 2025-06-30

**Authors:** Zhaohua Yang, Yunming Xu, Yanqiu Chu, Jinghao Li, Hong Wang

**Affiliations:** Pedietric Department of Shengjing Hospital, China Medical University, Shenyang 110004, China; yangzhaohua13@163.com (Z.Y.); xuym@sj-hospital.org (Y.X.); chuyanqiu2010@163.com (Y.C.); lijinghao2017@sina.com (J.L.)

**Keywords:** Kawasaki Disease, children, sST2, coronary artery lesion, myocardial damage, multi-organ damage

## Abstract

**Objectives:** Kawasaki Disease (KD) is an acute vasculitis associated with systemic inflammation. This study aimed to investigate the level and clinical significance of soluble ST2 (sST2) in children with KD. **Methods:** A retrospective analysis was conducted on 287 pediatric KD patients treated at the Pediatric Cardiology Department of Shengjing Hospital, China Medical University, from November 2021 to December 2022. Patients were stratified into subgroups based on the presence of myocardial damage (MD), coronary artery lesions (CAL), multi-organ involvement (MOD; ≥3 organs) and/or intravenous immunoglobulin-resistant KD (IVIG-R KD). In each group, we analyzed the correlation between sST2 levels and various laboratory parameters, including white blood cell count (WBC), hemoglobin (HB), platelet count (PLT), C-reactive protein (CRP), interleukin-6 (IL-6), erythrocyte sedimentation rate (ESR), N-terminal pro-brain natriuretic peptide (NT-pro BNP), D-dimer, and albumin (ALB). **Results:** Patients in the CAL group were significantly younger and predominantly male (*p* < 0.05). In the MD, CAL, MOD, and IVIG-R KD groups, levels of sST2, CRP, NT-pro BNP, and D-dimer were significantly higher than in their respective comparison groups (*p* < 0.05). sST2 showed weak positive correlations with WBC, CRP, IL-6, NT-pro BNP, and D-dimer, and weak negative correlations with HB and ALB (*p* < 0.05). sST2, HB, and IL-6 were identified as independent risk factors for MOD (*p* < 0.05). sST2 and HB were independent risk factors for IVIG-R KD (*p* < 0.05). Among acute-phase patients, four cases had sST2 levels > 200 ng/mL—all were classified as IVIG-R KD and MOD; three of these also developed coronary artery aneurysms (CAA). **Conclusions**: Elevated sST2 levels in the acute phase of KD may serve as a clinical indicator of IVIG-R KD, CAA, MOD, and MD.

## 1. Introduction

Exploring the relationship between elevated soluble growth stimulation expressed gene 2 protein (sST2) levels during the acute phase of Kawasaki Disease (KD) and the occurrence of coronary artery lesions (CAL), intravenous immunoglobulin-resistant KD (IVIG-R KD), and multi-organ damage (MOD) may contribute to timely diagnosis, targeted intervention, and the prevention of serious complications in children with KD. KD is an acute autoimmune vasculitis that primarily affects medium-sized arteries, with a predisposition towards coronary arteries. Among its complications, CALs are the most serious, with an incidence of 15–20% in untreated cases (defined as coronary artery Z score ≥ 2.5) [1]. KD has now become the leading cause of acquired heart disease in children worldwide. The use of IVIG has significantly reduced the incidence of CAL; however, IVIG resistance remains a critical challenge, as it is associated with a higher risk of coronary artery aneurysms (CAA) [2].

KD is characterized not only by coronary artery involvement, but also by systemic inflammation affecting multiple organs during the acute febrile phase. Clinical manifestations may include cardiac (myocarditis, pericarditis, valvulitis), gastrointestinal (abdominal pain, vomiting, diarrhea, gallbladder hydrops), hepatic (hepatitis), pulmonary (interstitial pneumonia), neurological (aseptic meningitis), renal (sterile pyuria) and pancreatic (pancreatitis). In rare but severe cases, KD Shock Syndrome (KDSS) or macrophage activation syndrome (MAS) may occur, posing life-threatening risks.

ST2, a member of the interleukin-1 receptor family, exists in two isoforms: the soluble form (sST2) and the transmembrane form (ST2L). First identified by Shin-ichi Tominaga in 1989, its cardiac relevance was later described by Richard Lee in 2002. In 2005, IL-33 was identified as the functional ligand for ST2 [3].

ST2 is produced by cardiomyocytes in response to mechanical stress, and plays a critical role in cardiovascular inflammation and remodeling. Under normal physiological conditions, ST2L binds IL-33 and initiates protective immune signaling pathways that regulate cardiomyocyte and fibroblast responses [4,5]. However, under pathological conditions, elevated sST2 acts as a decoy receptor, competitively binding IL-33 and blocking its interaction with ST2L. This inhibits cardioprotective signaling, thereby exacerbating myocardial injury, cell death, and fibrosis, and promoting disease progression. Since sST2 lacks the transmembrane and cytoplasmic domains of ST2L, it is secreted into the circulation and can be readily measured in blood samples. To date, most clinical research on sST2 has focused on heart failure and cardiovascular disease [6]. However, emerging evidence suggests that sST2 is also elevated during the acute phase of KD, which is characterized by necrotizing vasculitis, predominantly infiltrated by neutrophils and macrophages, along with elevated systemic inflammatory markers [7].

Given KD’s potential to cause long-term cardiovascular complications, understanding the role of sST2 as a biomarker may provide important insights into disease severity, the risk of IVIG resistance, and systemic involvement such as MOD.

## 2. Methods

### 2.1. Patients

A total of 287 children diagnosed with KD were included in this study. All patients were diagnosed and treated at the Department of Pediatric Cardiology, Shengjing Hospital, affiliated with the China Medical University, between November 2021 and December 2022.

### 2.2. sST2 Measurement

Serum sST2 levels were quantitatively measured using a double-antibody sandwich ELISA on a 96-well plate. A 2 mL venous blood sample was collected from each child prior to treatment with IVIG or glucocorticoids, and within 10 days of fever onset. The ELISA kit was supplied by Shanghai Ruidi Biotechnology Co., Ltd., and testing was performed using the Freedom Evolyzer-2100 automated immunoassay system (TECAN, Switzerland, Zurich).

### 2.3. Clinical and Laboratory Data Collection

In addition to sST2 testing, the following biochemical parameters were collected: complete blood count, including white blood cell count (WBC), hemoglobin (HB), and platelet count (PLT); inflammatory markers such as C-reactive protein (CRP), erythrocyte sedimentation rate (ESR) and interleukin-6 (IL-6); and cardiac biomarkers such as high-sensitivity troponin I, high-sensitivity troponin T and N-terminal pro-B-type natriuretic peptide (NT-proBNP). Other indicators include albumin (ALB), D-dimer, serum electrolytes, and alanine aminotransferase (ALT).

### 2.4. Imaging Data

Relevant imaging studies were undertaken, including electrocardiography (ECG), echocardiography (ECHO), electroencephalography (EEG), and chest computed tomography (CT). It has been reported that ST2 > 71 ng/mL in adults is regarded as the critical value of cardiac insufficiency. The higher the ST2, the more severe the cardiovascular damage. Four patients with sST2 levels > 200 ng/mL were identified and followed up through April 2025 to monitor clinical outcomes.

## 3. Definitions and Diagnostic Criteria

CAL: Diagnosed based on the 6th revised Japanese diagnostic guidelines using the Z-score system (http://raise.umin.jp/zsp/CALculator, accessed on 1 October 2020), with Z ≥ 2.5 considered diagnostic for CAL.

MOD: Defined as the involvement of three or more organ systems, including but not limited to cardiovascular (e.g., CAL, MD), hematologic (e.g., granulocytopenia, anemia, thrombocytopenia), electrolyte disturbances (e.g., hyponatremia, hypokalemia), neurological (e.g., sterile meningitis, facial nerve palsy), respiratory (e.g., interstitial pneumonia), hepatic (e.g., elevated ALT), genitourinary (e.g., urethritis), musculoskeletal (e.g., arthritis), and macrophage activation syndrome (MAS).

IVIG-R KD: Defined as a persistent or recurrent fever (≥38 °C) occurring 36–48 h after the completion of IVIG treatment at a dose of 2 g/kg, in the absence of any other identifiable cause [5].

### 3.1. Inclusion Criteria

Children diagnosed with KD were enrolled based on the 2017 American Heart Association (AHA) guidelines [8] and the sixth revised diagnostic criteria issued in Japan [9]. Only patients who received treatment and were available for follow-up were included.

### 3.2. Exclusion Criteria

Patients were excluded if they met any of the following conditions:

1. Duration of fever exceeded 10 days at the time of hospital admission;
2. sST2 testing was not performed, as measurements were only available on working days;
3. Administration of IVIG or corticosteroids within one month prior to sST2 testing.

### 3.3. Diagnostic Criteria for Myocardial Damage (MD)

MD was diagnosed based on the presence of elevated troponin I and/or troponin T levels above the normal upper limit; ECG abnormalities, including arrhythmias, prolonged P-R interval, non-specific ST-T changes, or low QRS voltage; ECHO findings, including left ventricular dilation, weakened ventricular wall motion, valve regurgitation, or reduced ejection fraction.

### 3.4. Patient Groups

A total of 287 patients with KD were categorized into the following groups based on laboratory, ECG, and ECHO results:

1. Myocardial Damage (MD)

Group A—MD present (*n* = 17)

Group B—MD absent (*n* = 270);

2. Coronary Artery Lesions (CAL)

Group C—CAL present (*n* = 48)

Group D—CAL absent (*n* = 239);

3. Multi-Organ Damage (MOD)

Group E—≥3 organs involved (*n* = 58)

Group F—<3 organs involved (*n* = 229);

4. IVIG-Resistant KD (IVIG-R KD)

Group G—IVIG-R KD present (*n* = 24)

Group H—IVIG-R KD absent (*n* = 263).

## 4. Statistical Analysis

All statistical analyses were conducted using SPSS version 27.0. Continuous variables conforming to a normal distribution were expressed as mean ± standard deviation (x ± s). Independent samples *t*-tests were used for comparisons with equal variances. Welch’s *t*-tests were used for unequal variances. Data with a skewed distribution were expressed as median (interquartile range, IQR), and the Mann–Whitney U test was applied. Categorical variables were presented as frequency and percentage, and compared using the Chi-square test. Binary logistic regression analysis was used to identify independent risk factors for MOD and IVIG-R KD. The receiver operating characteristic (ROC) curve was employed to evaluate the predictive value of sST2. Spearman correlation analysis was used to assess the relationships between sST2 levels and laboratory indicators. A *p*-value < 0.05 was considered statistically significant.

### Ethical Approval

This study was reviewed and approved by the Ethics Committee of Shengjing Hospital, China Medical University (Approval No. 2023PS152J).

## 5. Results

### 5.1. The Age and Gender Distribution of Children with KD

Among the 287 children with KD, 213 (72.20%) were under 3 years old, and the male-to-female ratio was 1.58:1. The percentage of males and the age were both lower in the KD with CAL group (*p* < 0.05) (Table 1).

### 5.2. Comparison of sST2 Levels Among Different Groups

The sST2 levels in groups A, C, E and G were significantly higher than those in groups B, D, F, and H (*p* < 0.05) (Table 2).

### 5.3. Comparison of Other Indicators Among Different Groups

The levels of CRP, NT-pro BNP and D-dimer in group A, C, E, and G were higher than those in groups B, D, F, and H, respectively (*p* < 0.05) (Table 3).

### 5.4. Correlation Analysis Between sST2 and Other Indicators

The correlation coefficient r was calculated using Spearman correlation analysis. A correlation was considered weak when 0.3 ≤ |r| < 0.5. sST2 had a weak positive correlation with WBC, CRP, IL-6, NT-pro BNP, and D-dimer, and a weak negative correlation with HB and ALB. There was no correlation between sST2 and ESR or PLT (Table 4).

### 5.5. KD Combined with MD

Based on the differences observed between group A (KD with MD) and group B (KD without MD), as shown in Table 3, the following variables were included as independent predictors in a univariate binary logistic regression analysis: sST2, WBC, PLT, CRP, IL-6, D-dimer, and NT-pro BNP. The analysis reveals that elevated levels of sST2, WBC, and CRP were significant factors promoting MD in patients with KD (*p* < 0.05). However, when these variables were further analyzed using multivariate logistic regression, none of them remained statistically significant, suggesting that no single factor independently predicted myocardial damage after adjusting for covariates (Table 5).

### 5.6. KD Combined with CAL

Based on the differences observed between group C (with CAL, CAL) and group D (without CAL) in Table 3, the following variables were included as independent predictors in a univariate binary logistic regression analysis: sST2, HB, PLT, CRP, NT-pro BNP, D-dimer, ALB, age, and gender (coded as male = 1). The models for sST2, HB, PLT, CRP, ALB, age, and gender demonstrated good fit, indicating reliable performance.

The univariate analysis shows that elevated sST2, PLT, and CRP, as well as decreased HB and ALB, younger age, and male gender, were all significantly associated with an increased risk of coronary artery lesions (*p* < 0.05). However, in the subsequent multivariate logistic regression analysis, only male gender remained a statistically significant independent risk factor for coronary artery damage (*p* < 0.05) (Table 6).

### 5.7. KD Combined with MOD

Based on the differences between group E (three or more organs involved, MOD) and group F (less than three organs involved) shown in Table 3, the following variables were included as independent predictors in a univariate binary logistic regression analysis: sST2, WBC, HB, PLT, CRP, IL-6, NT-pro BNP, D-dimer, and ALB.

The models constructed with sST2, WBC, HB, PLT, IL-6, and D-dimer demonstrated good fit and were considered valid. These variables were subsequently included in a multivariate binary logistic regression analysis, which revealed that elevated sST2 and IL-6, along with reduced HB, were independent risk factors for multi-organ involvement in children with KD (*p* < 0.05) (Table 7).

**Figure 1:**
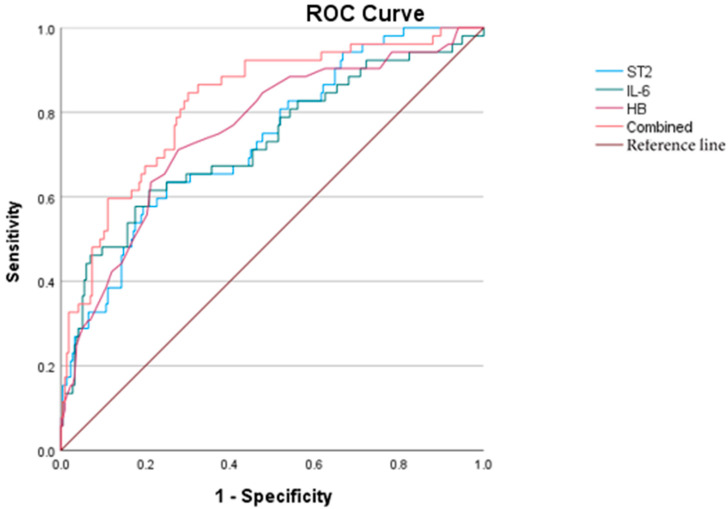
ROC curves of sST2, IL-6, HB, and the combined diagnosis for KD with MOD.

To further evaluate the predictive values of key variables for MOD in KD, a receiver operating characteristic (ROC) curve analysis was performed. The area under the curve (AUC) values for sST2, IL-6, and HB were 0.735, 0.728, and 0.756, respectively. When these three markers were combined, the AUC increased to 0.823, suggesting improved predictive performance. The optimal cut-off value for sST2 in predicting MOD was determined to be 51.264 ng/mL (Figure 1).

### 5.8. IVIG-R KD

Binary logistic regression analysis demonstrated that models incorporating sST2, HB, CRP, IL-6, ALB, and IVIG-R KD exhibited good fit. Among these, sST2 and HB were identified as independent risk factors for IVIG-R KD (*p* < 0.05) (Table 8).

**Figure 2:**
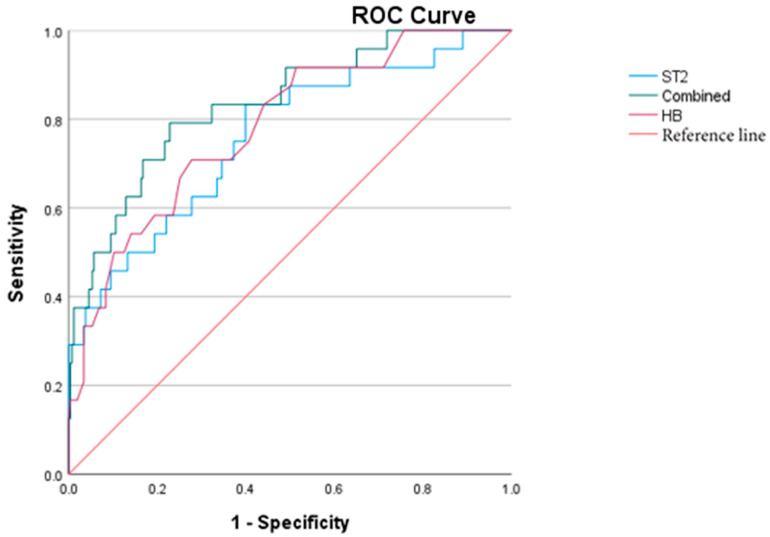
ROC curves of sST2, HB, and the combined prediction of IVIG-R KD.

To further assess the predictive value of sST2 and HB for IVIG-R KD, a ROC curve analysis was performed. The AUCs for sST2 and HB were 0.760 and 0.783, respectively. When combined, the AUC increased to 0.835, demonstrating improved predictive performance. Notably, elevation in sST2 occurred earlier in the disease course than the decline in HB. The optimal cut-off value of sST2 for predicting IVIG-R KD was determined to be 43.412 ng/mL (Figure 2).

### 5.9. Clinical Data of Four Cases with ST2 > 200 ng/mL

Among acute-phase patients, four cases had sST2 levels > 200 ng/mL—all were classified as IVIG-R KD and MOD; three of these also developed CAA (Table 9).

## 6. Discussion

Soluble ST2 (sST2), a member of the interleukin-1 (IL-1) receptor family, has emerged as a critical biomarker in cardiovascular and inflammatory diseases. Its specific ligand, interleukin-33 (IL-33), also part of the IL-1 family, forms the IL-33/ST2 axis, a signaling pathway increasingly recognized for its role in immune regulation and cardiovascular pathology. Since its initial identification by Shin-ichi Tominaga in 1989 and its association with cardiac disease described by Richard Lee’s group in 2002, sST2 has been extensively studied for its predictive value in heart failure, myocardial fibrosis, and cardiac remodeling [10,11,12].

There are two forms of ST2—transmembrane ST2L, which binds IL-33 and activates protective signaling pathways, and soluble ST2 (sST2), which acts as a decoy receptor. Under physiological conditions, IL-33/ST2L signaling inhibits cardiac hypertrophy and fibrosis, contributing to myocardial protection [11]. However, elevated sST2 levels competitively inhibit IL-33/ST2L binding, disrupting these protective mechanisms and allowing disease progression. Unlike NT-pro BNP, sST2 levels are not influenced by renal function, making this a potentially superior biomarker in settings of volume depletion or pre-renal insufficiency common during the acute phase of KD [12,13].

Our study demonstrates that sST2 is significantly elevated in patients with CAA, consistent with prior research [4]. Notably, three patients with CAA exhibited elevated sST2 despite normal NT-pro BNP levels, highlighting sST2′s sensitivity and the early rise in acute inflammation. This may be attributed to necrosis of the mid-coronary artery, which triggers the release of sST2.

Furthermore, patients with MOD or IVIG-R KD also exhibited elevated sST2, though to a lesser degree than those with CAA. In our cohort, four patients had sST2 levels exceeding 200 ng/mL, and all developed both IVIG-R KD and MOD. Their clinical courses illustrate the severity of illness and the extent of systemic involvement.

Case 1: Eight-organ involvement, cardiogenic shock, heart failure (recovery in 10 days), aseptic meningitis (recovered in 3 months).

Case 2: Six-organ involvement, leukemia-like reaction (recovered in 3 weeks), middle CAA (resolved within 2 years).

Case 3: Persistent fever (>3 weeks), six-organ involvement, giant CAA (resolved in 2 years).

Case 4: Fever for 22 days, seven-organ involvement, persistent large CAA (ongoing follow-up through April 2025).

These cases suggest that a marked elevation in sST2 is associated with severe inflammation and poor prognosis, consistent with findings from sepsis studies, where higher sST2 levels were linked to shock, multi-organ failure, and mortality [3].

Logistic regression analysis in our study identified elevated sST2 and IL-6 and low HB as independent risk factors for MOD. ROC curve analysis showed that HB reduction had the highest predictive value, followed by sST2. However, sST2 elevation occurred earlier in the disease course than HB reduction, suggesting that sST2 may serve as an early warning marker for severe KD. The combination of sST2, IL-6, and HB yielded the best predictive accuracy for MOD.

For IVIG-R KD, multivariate analysis also showed that sST2 and HB were independent predictors, with sST2 having superior predictive value. These findings emphasize the clinical utility of sST2 not only in identifying coronary complications, but also in forecasting treatment resistance and systemic involvement.

Additionally, our study corroborated previous findings that CAL is more common in younger, male patients, and that MD is associated with elevated sST2. However, in multivariate analysis, sST2 was not a significant independent predictor of CAL or MD, potentially due to sample size limitations and the short study duration, indicating the need for further investigation.

The relevance of the IL-33/ST2 axis extends beyond KD. Emerging literature has drawn parallels between KD and Multisystem Inflammatory Syndrome in Children (MIS-C) associated with COVID-19. MIS-C shares features with KD, including fever, rash, conjunctivitis, mucosal changes, and multi-organ involvement [14,15,16]. IL-33 signaling plays a pivotal role in immune activation in MIS-C, involving MAPK, NF-κB, and MyD88 pathways, contributing to a cytokine storm and MOD [17]. The elevation of sST2 in MIS-C and KD likely reflects this shared pathophysiological basis.

Importantly, persistent CAA in KD patients poses long-term risks. Although the precise prognostic significance is not fully understood, autopsy findings in patients with long-standing CAA have shown extensive myocardial fibrosis, likely due to chronic coronary inflammation and ischemia. Thus, the long-term monitoring of sST2 may provide prognostic insight into cardiac remodeling and guide post-acute care.

## 7. Conclusions

In summary, elevated sST2 levels during the acute phase of KD are strongly associated with IVIG resistance, coronary artery involvement, multi-organ damage, and myocardial injury. sST2 may serve as an early biomarker for identifying severe or atypical KD cases, guiding timely and aggressive intervention. Its independence from renal function and early rise enhance its clinical utility over traditional markers like NT-pro BNP.

What is particularly promising is the growing evidence that the IL-33/sST2 axis may represent a therapeutic target in KD vasculitis [4]. However, due to the limited sample size and a lack of non-KD febrile controls, further studies with larger cohorts and comparative groups are needed. The long-term follow-up of KD patients with persistent CAA and elevated sST2 levels may provide valuable insight into disease progression and outcomes.

## Figures and Tables

**Table 1:** General information.

	Groups	Age (year)	Male (%)	*P_year_*	*P_gender_*
MD	A (17)	2.0 (0.6~3.0)	9 (52.94)	0.061	0.464
	B (270)	2.5 (1.5~4.0)	167 (61.85)		
CAL	C (48)	1.9 (0.8~2.7)	40 (83.33)	0.003	<0.001
	D (239)	2.5 (1.5~4.0)	136 (56.90)		
MOD	E (58)	2.7 (1.0~4.0)	36 (62.07)	0.849	0.896
	F (229)	2.4 (1.4~4.0)	140 (61.14)		
IVIG-R KD	G (24)	2.8 (1.8~5.0)	17 (70.83)	0.109	0.318
	H (263)	2.4 (1.4~4.0)	159 (60.46)		

MD, myocardial damage; CAL, coronary artery lesion; MOD, multi-organ damage; IVIG-R KD, IVIG-resistant KD.

**Table 2:** Comparison of sST2 levels among different groups.

	Groups	sST2 (ng/mL)	*Z*	*p*
MD	A (17)	55.53 (41.97~120.58)	−3.150	0.002
	B (270)	38.28 (27.25~57.60)		
CAL	C (48)	42.82 (32.24~71.78)	−2.086	0.037
	D (239)	38.35 (27.14~57.46)		
MOD	E (58)	59.58 (37.47~96.14)	−5.380	<0.001
	F (229)	37.49 (26.33~51.83)		
IVIG-R KD	G (24)	65.67 (43.96~183.66)	−4.214	<0.001
	H (263)	37.73 (27.29~55.62)		

MD, myocardial damage; CAL, coronary artery lesion; MOD, multi-organ damage; IVIG-R KD, IVIG-resistant KD.

**Table 3:** Comparison of other indicators among different groups.

	*P*_WBC_	*P*_HB_	*P*_PLT_	*P*_CRP_	*P*_IL-6_	*P*_ESR_	*P*_*pro*-BNP_	*P*_D-dimer_	*P*_ALB_
A vs. B	0.001	0.134	0.046	0.018	0.002	0.348	<0.001	0.003	0.072
C vs. D	0.37	0.001	0.011	0.039	0.076	0.933	0.037	0.005	0.032
E vs. F	0.002	<0.001	0.002	<0.001	<0.001	0.772	<0.001	<0.001	<0.001
G vs. H	0.032	<0.001	0.05	<0.001	0.001	0.288	0.009	0.002	<0.001

WBC, white blood cell; HB, hemoglobin; PLT, platelet; CRP, C-reactive protein; ESR, erythrocyte sedimentation rate; IL-6, interleukin 6; pro-BNP, pro-B-type natriuretic peptide; ALB, serum albumin.

**Table 4:** Correlation analysis between sST2 and other indexes.

	Indexes	r	Sig.	95% Confidence Interval (CI)	
				Lower Limit	Upper Limit
sST2	WBC	0.301	<0.001	0.188	0.405
	HB	−0.333	<0.001	−0.434	−0.222
	PLT	0.196	<0.001	0.079	0.308
	CRP	0.412	<0.001	0.308	0.506
	IL-6	0.456	<0.001	0.352	0.548
	ESR	0.105	0.08	−0.016	0.223
	NT-pro BNP	0.419	<0.001	0.315	0.514
	D-dimer	0.367	<0.001	0.258	0.467
	ALB	−0.403	<0.001	−0.499	−0.299

WBC, white blood cell; HB, hemoglobin; PLT, platelet; CRP, C-reactive protein; ESR, erythrocyte sedimentation rate; IL-6, interleukin 6; pro BNP, pro-B-type natriuretic peptide; ALB, serum albumin.

**Table 5:** Logistic regression analysis of KD combined with MD.

Factor	Influence Factor	B	SE	Wald	*p*	OR	95%CI	
							Lower Limit	Upper Limit
single	sST2	0.011	0.004	7.043	0.008	1.011	1.003	1.020
	WBC	0.099	0.035	7.788	0.005	1.104	1.030	1.183
	CRP	0.012	0.004	10.034	0.002	1.012	1.004	1.019
multi	sST2	0.004	0.006	0.544	0.461	1.004	0.993	1.015
	WBC	0.051	0.044	1.324	0.250	1.052	0.965	1.147
	CRP	0.007	0.005	1.909	0.167	1.007	0.997	1.017

sST2, soluble ST2; WBC, white blood cell; CRP, C-reactive protein.

**Table 6:** Logistic regression analysis of KD combined with CAL.

Factor	Influence Factor	B	SE	Wald	*p*	OR	95%CI	
							Lower Limit	Upper Limit
single	sST2	0.010	0.004	7.680	0.006	1.010	1.003	1.017
	HB	−0.049	0.015	11.031	<0.001	0.952	0.924	0.980
	PLT	0.003	0.001	10.512	0.001	1.003	1.001	1.005
	CRP	0.009	0.003	11.073	<0.001	1.009	1.004	1.014
	ALB	−0.120	0.046	6.804	0.009	0.887	0.811	0.971
	age	−0.253	0.103	6.024	0.014	0.776	0.634	0.950
	gender	1.332	0.409	10.612	0.001	3.787	1.700	8.437
multi	sST2	0.003	0.005	0.284	0.594	1.003	0.993	1.012
	HB	−0.013	0.019	0.505	0.477	0.987	0.951	1.024
	PLT	0.002	0.001	2.889	0.089	1.002	1.000	1.004
	CRP	0.005	0.004	1.411	0.235	1.005	0.997	1.012
	ALB	−0.065	0.059	1.204	0.272	0.937	0.834	1.053
	age	−0.174	0.110	2.503	0.114	0.840	0.677	1.043
	gender	1.430	0.435	10.804	0.001	4.179	1.781	9.803

sST2, soluble ST2; HB, hemoglobin; PLT, platelet; CRP, C-reactive protein; ALB, serum albumin.

**Table 7:** Logistic regression analysis of KD combined with MOD.

Factor	Influence Factor	B	SE	Wald	*p*	OR	95%CI	
							Lower Limit	Upper Limit
single	sST2	0.025	0.005	24.92	<0.001	1.025	1.015	1.035
	WBC	0.078	0.026	8.91	0.003	1.081	1.027	1.137
	HB	−0.085	0.016	28.99	<0.001	0.918	0.890	0.947
	PLT	0.002	0.001	7.97	0.005	1.002	1.001	1.004
	IL-6	0.005	0.001	22.39	<0.001	1.005	1.003	1.008
	D-dimer	0.001	0.000	16.44	<0.001	1.001	1.001	1.002
multi	sST2	0.013	0.005	6.01	0.014	1.013	1.003	1.024
	HB	−0.067	0.021	10.65	0.001	0.935	0.898	0.974
	IL-6	0.003	0.001	5.79	0.016	1.003	1.001	1.006
	WBC	−0.021	0.040	0.28	0.600	0.979	0.905	1.059
	PLT	0.001	0.001	0.51	0.477	1.001	0.998	1.003

sST2, soluble ST2; HB, hemoglobin; IL-6, interleukin 6; WBC, white blood cell; PLT, platelet.

**Table 8:** Logistic regression analysis of IVIG-R KD.

Factor	Influence Factor	B	SE	Wald	*p*	OR	95%CI	
							Lower Limit	Upper Limit
single	sST2	0.026	0.005	24.142	<0.001	1.025	1.016	1.037
	HB	−0.107	0.022	23.786	<0.001	0.899	0.861	0.938
	CRP	0.017	0.003	24.584	<0.001	1.017	1.010	1.024
	IL-6	0.003	0.001	6.239	0.013	1.003	1.001	1.005
	ALB	−0.243	0.069	12.369	<0.001	0.785	0.685	0.898
multi	sST2	0.017	0.006	7.987	0.005	1.017	1.005	1.029
	HB	−0.062	0.027	5.354	0.021	0.940	0.892	0.991
	CRP	0.006	0.005	1.143	0.285	1.006	0.995	1.016
	IL-6	0.000	0.001	0.416	0.519	1.000	0.999	1.002
	ALB	0.059	0.086	0.477	0.490	1.061	0.897	1.256

sST2, soluble ST2; HB, hemoglobin; CRP, C-reactive protein; IL-6, interleukin 6; ALB, albumin.

**Table 9:** Clinical data of four cases with sST2 > 200.

Case	Gender	Age	sST2 (ng/mL)	Fever	Treatment	MOD
1# 19 kg 107 cm	F	3.5 y	>200	Admission 7 d Regressive10 d	IVIG 4 g/kg Dex5 mg*2 d Methylmethicone: 2 mg/kg*7 d 1.5 mg/kg*7 d 1 mg/kg*1 d Prednisone Po 14 d ALB IV 40 g	Cardiogenic shock Acute heart failure Hypoproteinemia (27.1 g/L) Hypokalemia, hyponatremia, Pneumonia Aseptic encephalitis (EEG 2–3 Hz) Localized peritonitis Thrombocytopenia
2# 13.5 kg 102 cm	M	3 y	>200	Admission 9 d Regressive20 d	IVIG 2 g/kg Methylmethicone: 20 mg/kg*3 d 2 mg/kg*3 d 1 mg/kg*1 d Prednisone Po 7 d ALB IV 10 g	CAA: LM4.7 mm, Z = 6.07, 3 m recovered Liver damage (ALT 95 U/L) Hypoproteinemia (24 g/L) Leukemoid reaction Aseptic encephalitis (EEG 5–7 Hz) Pneumonia,
3# 9.3 kg 82 cm	M	23 m	285.4	Admission 5 d Regressive27 d	IVIG 4 g/kg Methylmethicone 20 mg/kg*3 d 10 mg/kg*3 d 2 mg/kg*4 d 1 mg/kg*10 d Prednisone Po 10 d TNF inhibitor 5 mg/kg ALBI V 70 g	CAA: LM5.6 mm (Z = 11.1) RCA6.5 mm (Z = 12) Liver damage (ALT 434 U/L) Hypoproteinemia (24.2 g/L) Aseptic encephalitis (CSF:WBC66, Pro 0.56) Pleural effusion Moderate anemia (HGB = 76 g/L)
4# 29 kg 130 cm	F	9 y	287.2	Admission 6 d Regressive 22 d	IVIG 3 g/kg Methylmethicone: 2 mg/kg*6 d 1 mg/kg*7 d 0.7 md/kg*3 d Prednison Po 10 d ALBIV 60 g CTX 2 mg/kg IV	CAA: LAD 6.9 mm (Z = 7.63) persist RCA7.7 mm (Z = 10.63) persist Hypoproteinemia (20.6 g/L) Aseptic encephalitis (EEG 4–7 Hz) Knee joint effusion Granulocytopenia Hyponatremia, Moderate anemia (HGB = 86 g/L)

MOD, multi-organ damage; Dex, dexamethasone; ALB, albumin; CAA, coronary artery aneurysm; LM, left main coronary artery; RCA, right coronary artery; CSF, Cerebrospinal fluid; WBC, white blood cell.

## Data Availability

Because it involves patient privacy, especially for those with CAA, these clinical data cannot be made public.
